# Supplementary material for: High resolution spatial profiling of kidney injury and repair using RNA hybridization-based in situ sequencing
Source: Nat Commun. 2024 Feb 15;15:1396. doi: 10.1038/s41467-024-45752-8 (PMC10869771; doi:10.1038/s41467-024-45752-8)
Supplement: Supplementary file 1 — Supplementary Information [file 41467_2024_45752_MOESM1_ESM.pdf]

Supplementary Information

High resolution spatial profiling of kidney injury and repair using RNA hybridization-based in situ sequencing

Haojia Wu, Eryn E. Dixon, Qiao Xuanyuan, Juanru Guo, Yasuhiro Yoshimura, Chitnis Debashish, Anezka Niesnerova, Hao Xu, Morgane Rouault and Benjamin D. Humphreys

Supplementary Table 1 | **Summary of cell count**. Total number of nuclei were counted based on the DAPI staining. Total cells are the high quality cells for downstream analysis after cell filtering.

Sample	Total nuclei	Total cells
Sham_male	123769	78392
Hour4	119572	114658
Hour12	100781	93990
Day2	102392	101702
Week6	135106	119666
Sham_female	117814	88961

Supplementary Table 2 | **Comparison of the spatial analysis tools**.

Analysis	CellScopes.jl	Seurat	Giotto	Squidpy
Platform	Julia	R	R + Python	Python
New spatial datatype	Yes	No	No	No
Grid system for FOV selection	Yes	No	No	No
Gene on polygons	Yes	Yes	Yes	No
Cell polygons	Yes	Yes	Yes	No
Cell proximity analysis	Yes	No	Yes	Yes
Whole tissue cell scanning	Yes	No	No	No
Gene imputation	Yes	No	No	No

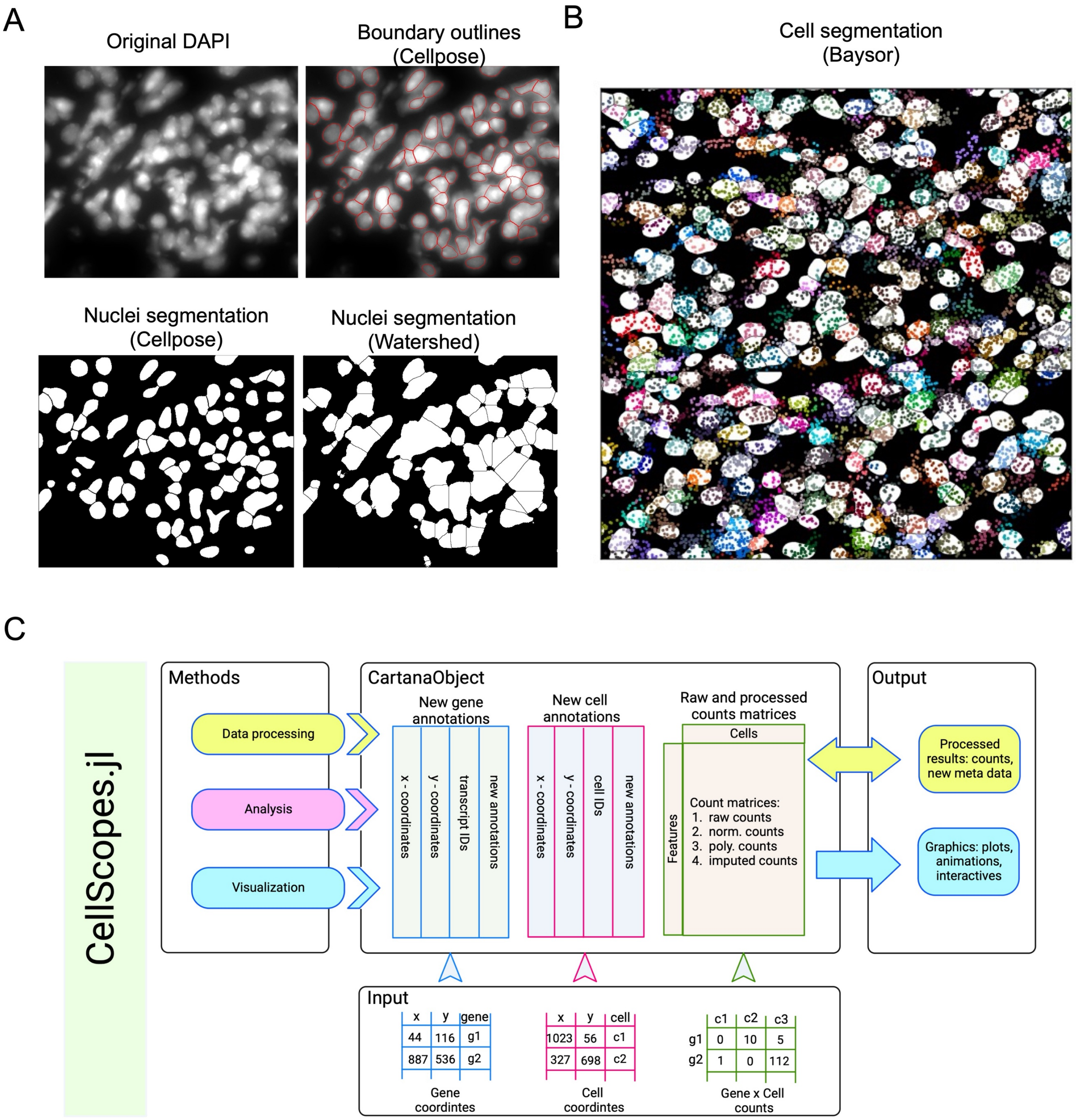

Supplementary Figure 1. Cell segmentation and data visualization
(**A**) Nucleus segmentation by CellPose and Watershed. (**B**) Cell segmentation by Baysor using the nucleus segmentation as prior information. (**C**) Data structure for dRNA HybISS data analysis constructed by CellScopes.

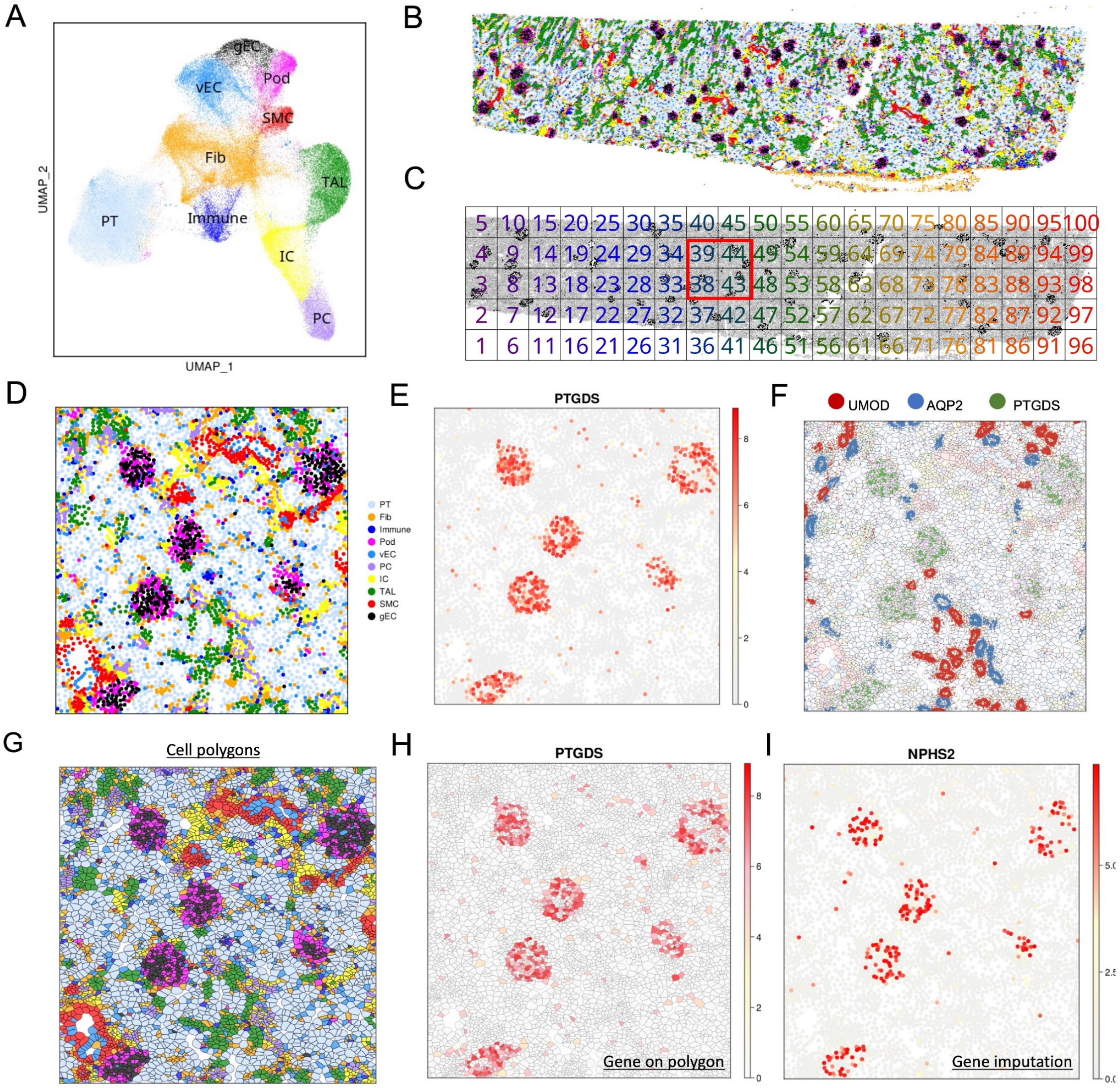

Supplementary Figure 2. A public human kidney Xenium dataset analyzed by CellScopes.jl. (**A**) Cell clustering and cell type annotation. (**B**) Spatial projection of the cell labels. (**C**) A grid system to facilitate the selection of field of view (FOV). (**D**) Zoom in the selected FOV to visualize the cell labels. (**E**) Zoom in the selected FOV to visualize the gene expression. (**F**) Zoom in the selected FOV to visualize the transcript distribution of multiple genes. (**G**) Visualizing cells with cell boundary in polygon format. (**H**) Visualizing genes with cell boundary in polygon format. (**I**) Visualizing genes after gene imputation.

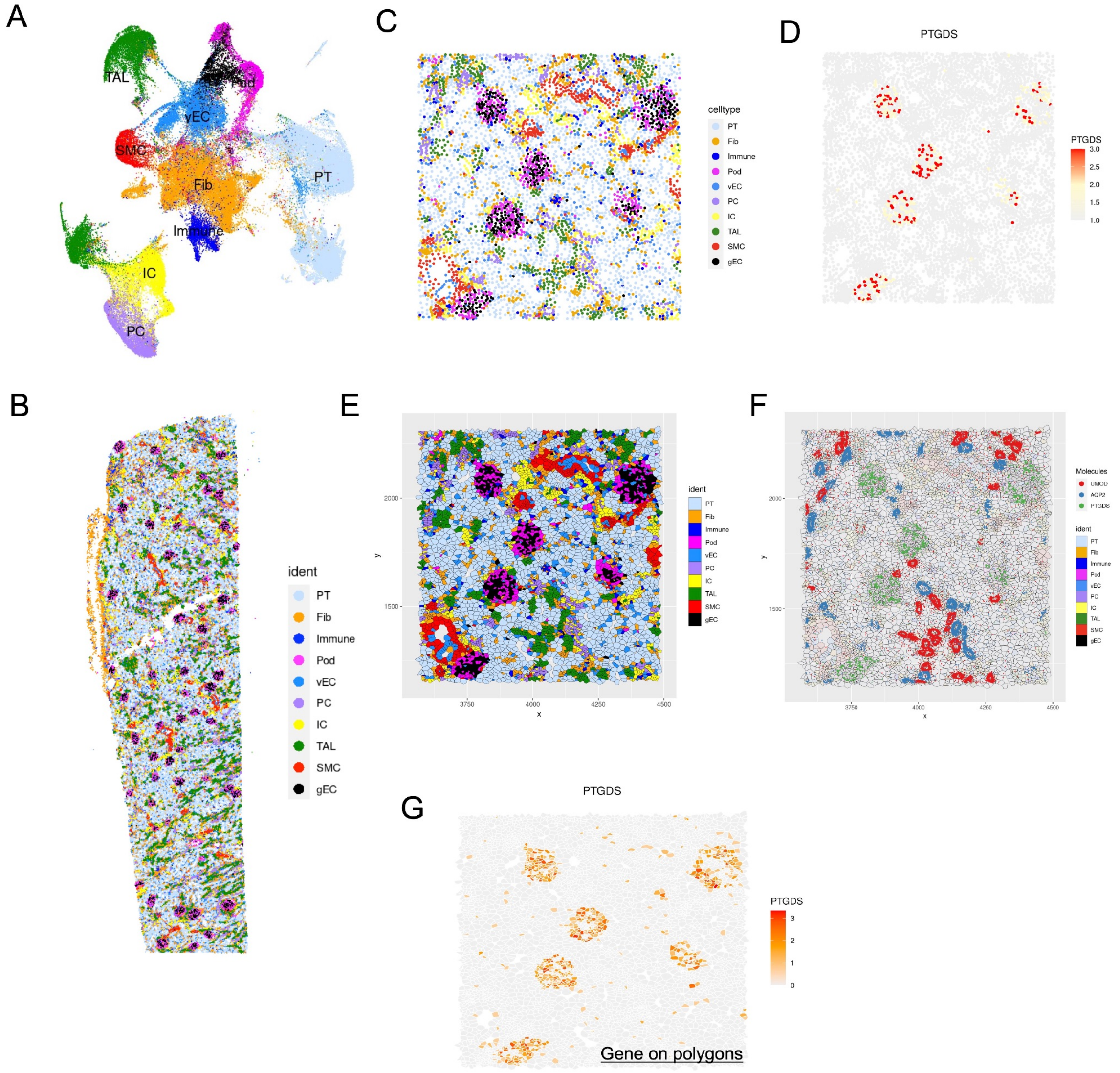

Supplementary Figure 3. A public human kidney Xenium dataset analyzed by Seurat V5 in R. (A) Cell clustering and cell type annotation. (**B**) Spatial projection of the cell labels. (**C**) Zoom in the selected FOV to visualize the cell labels. (**D**) Zoom in the selected FOV to visualize the gene expression. (**E**) Zoom in the selected FOV to visualize the transcript distribution of multiple genes. (**F**) Visualizing cells with cell boundary in polygon format. (**G**) Visualizing genes with cell boundary in polygon format.

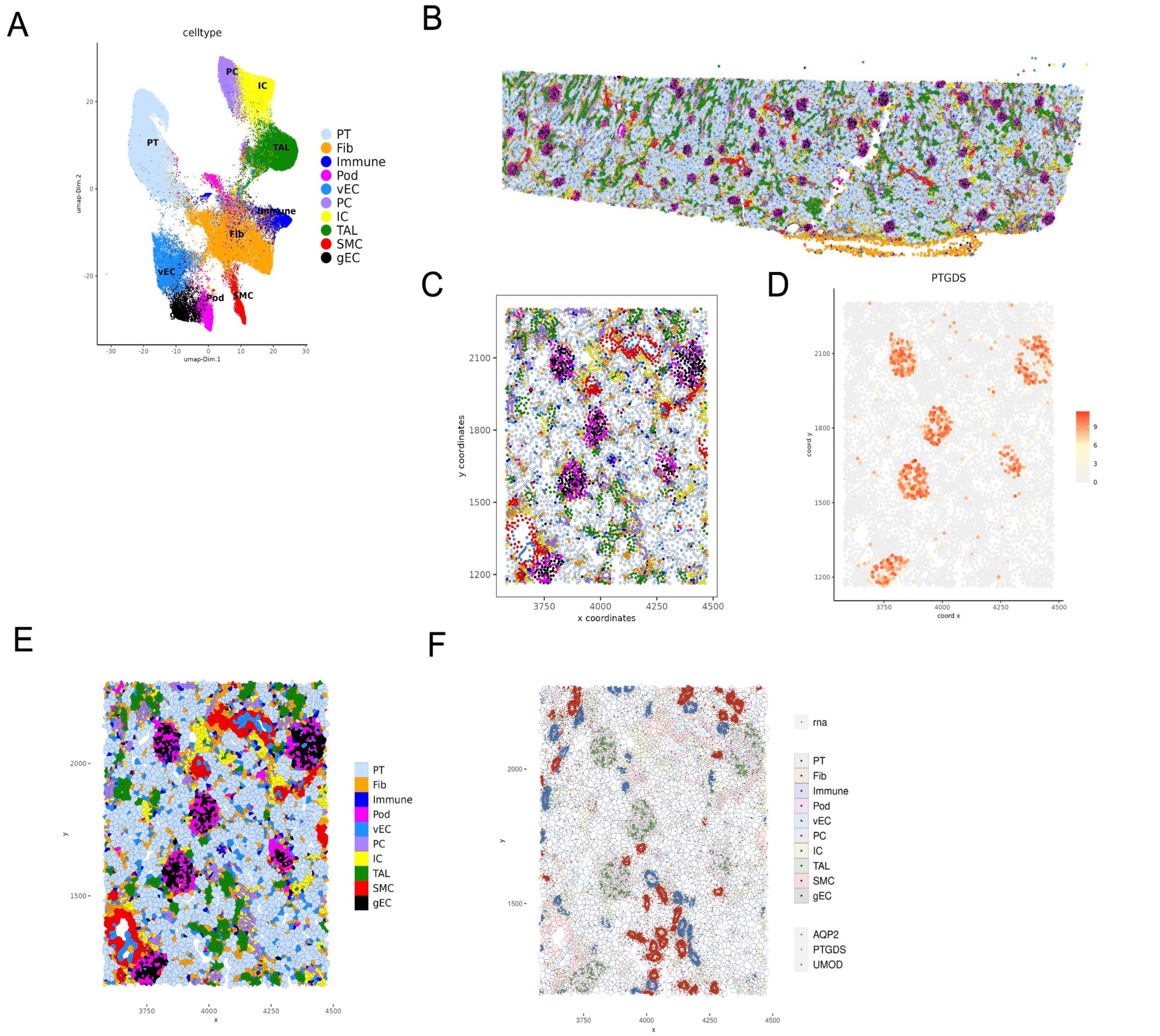

Supplementary Figure 4. A public human kidney Xenium dataset analyzed by Giotto in R and python. (**A**) Cell clustering and cell type annotation. (**B**) Spatial projection of the cell labels. (**C**) Zoom in the selected FOV to visualize the cell labels. (**D**) Zoom in the selected FOV to visualize the gene expression. (**E**) Zoom in the selected FOV to visualize the transcript distribution of multiple genes. (**F**) Visualizing cells with cell boundary in polygon format.

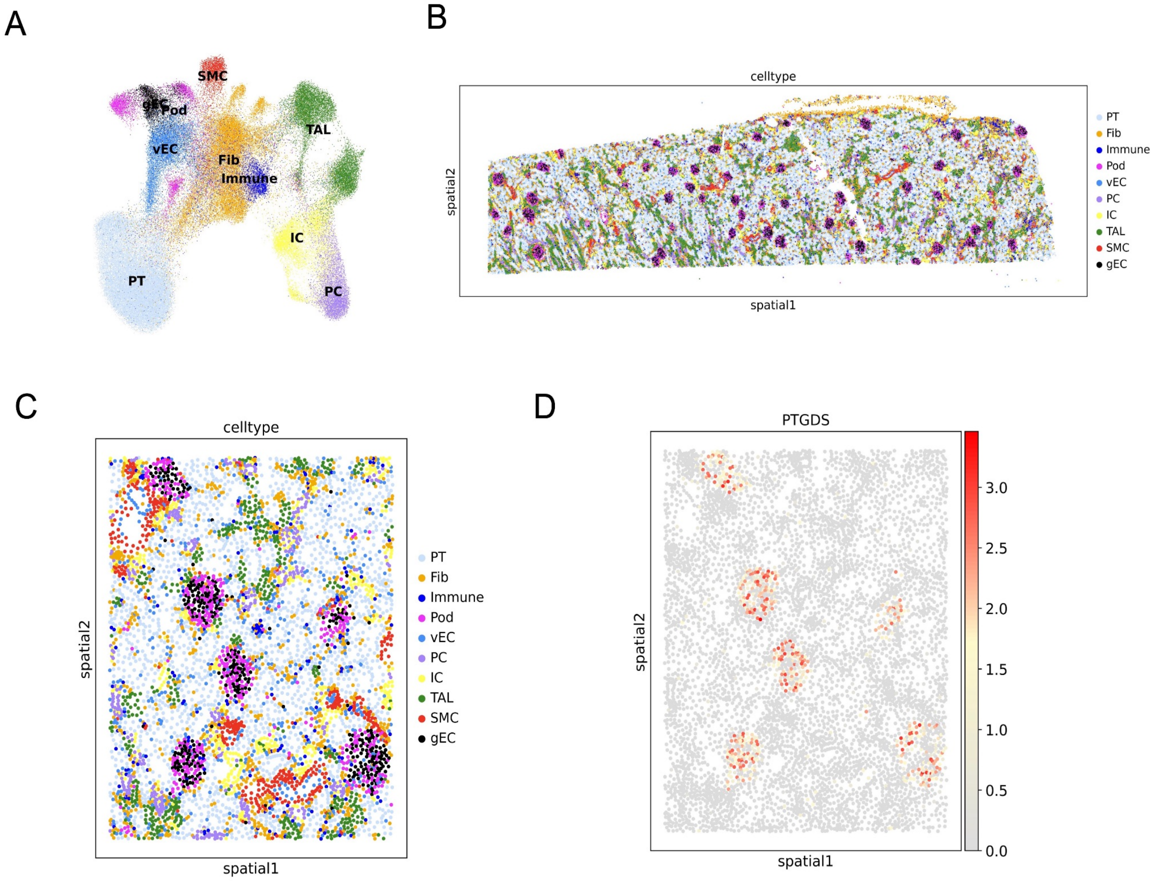

Supplementary Figure 5. A public human kidney Xenium dataset analyzed by Squidpy in python. (**A**) Cell clustering and cell type annotation. (**B**) Spatial projection of the cell labels. (**C**) Zoom in the selected FOV to visualize the cell labels. (**D**) Zoom in the selected FOV to visualize the gene expression.

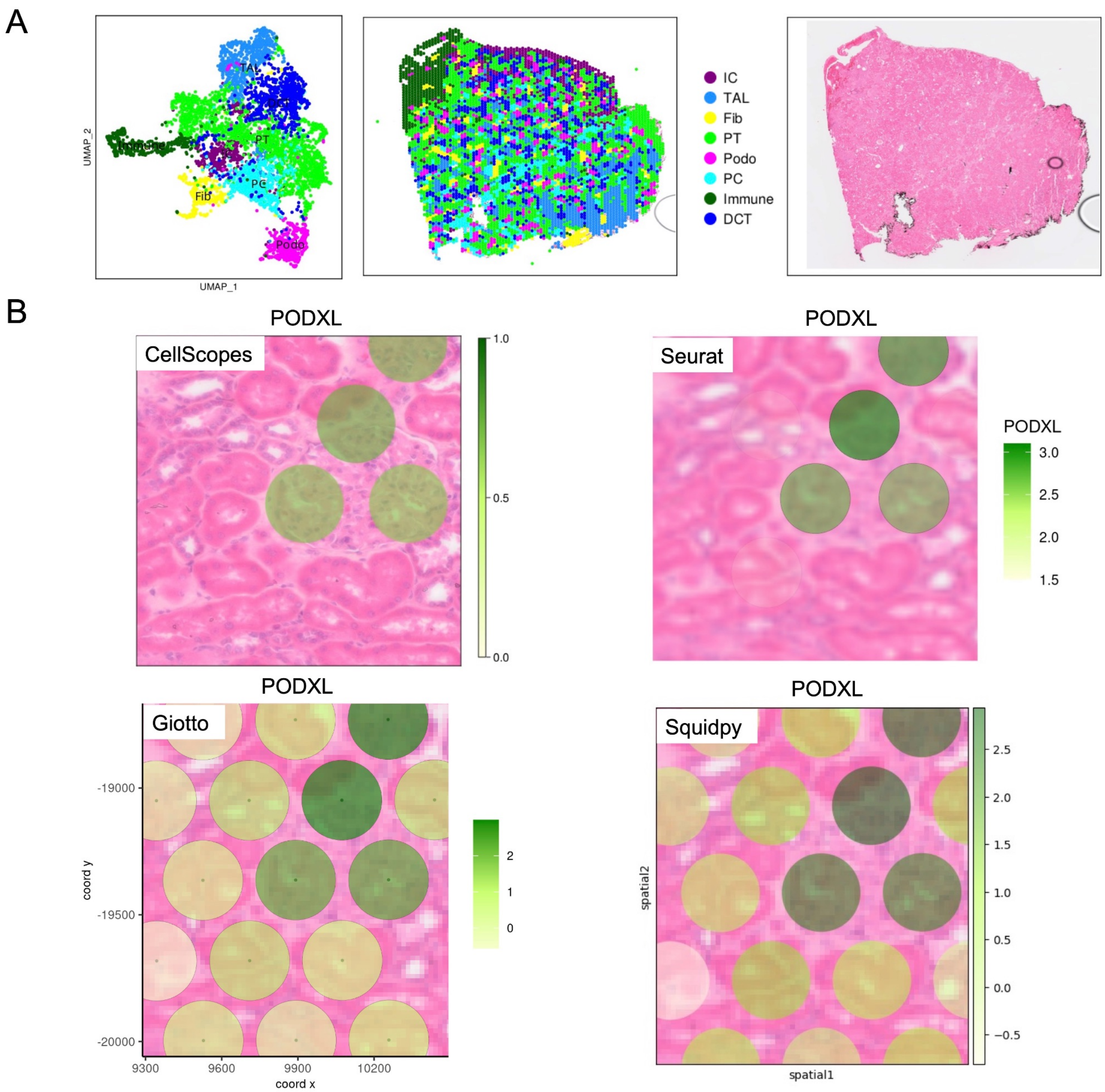

Supplementary Figure 6. A public human kidney Visium dataset analyzed by CellScopes, Seurat, Giotto and Squidpy. (**A**) Cell clustering and cell type annotation by CellScopes. (**B**) Visualization of PODXL expression in the same FOV using CellScopes, Seurat, Giotto, and Squidpy.

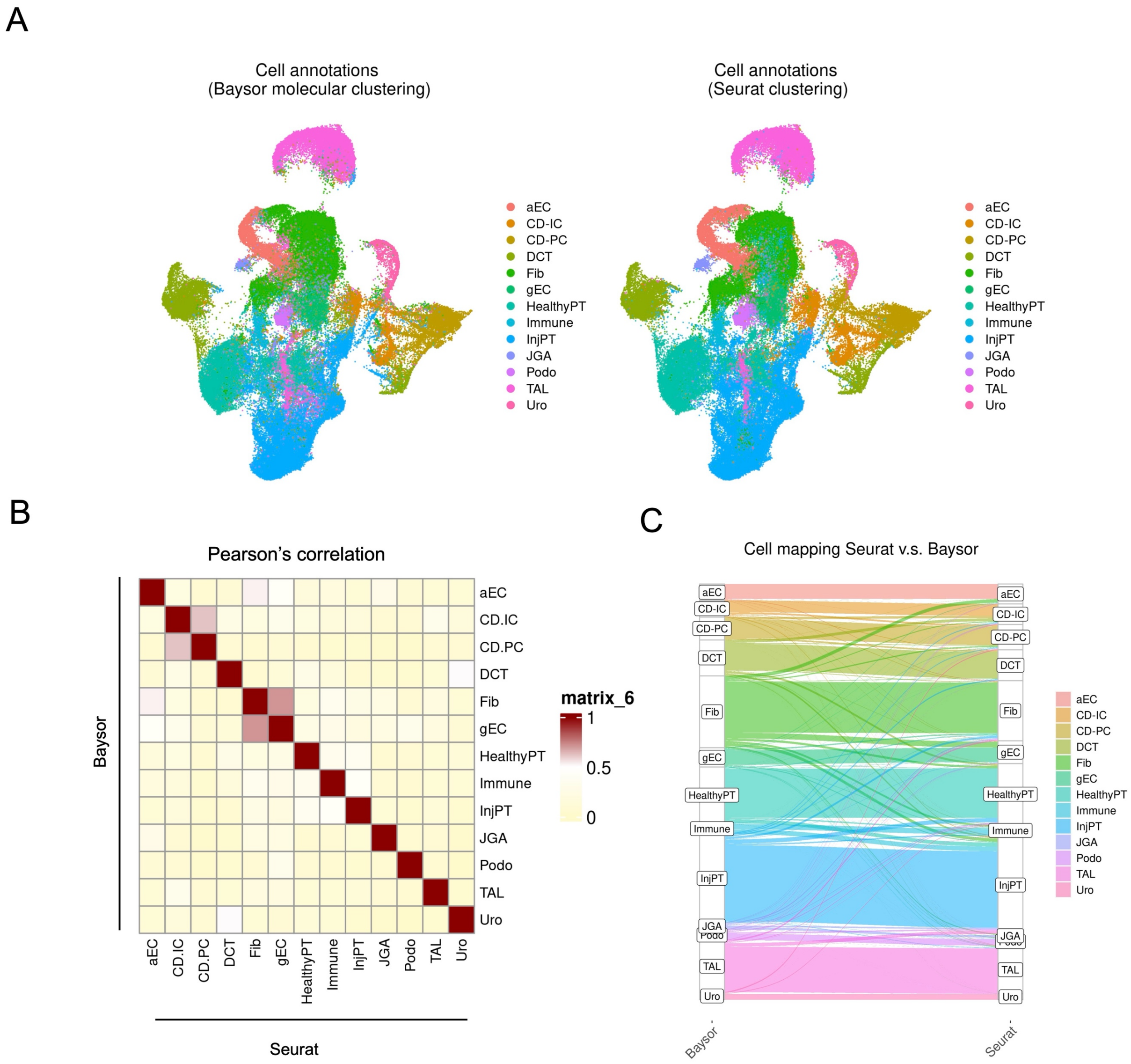

Supplementary Figure 7. Comparison of cell annotations using Baysor and Seurat. (**A**) UMAP projection of cells. Cells are projected in a UMAP space, color-coded according to their respective cell type annotations derived from Baysor or Seurat. (**B**) Cell type correlations. The correlation analysis based on gene expression across cell types, as categorized by Baysor and Seurat. (**C**) Cell type mapping. A cell map showing how cells from each cell type identified by Baysor correspond to the cell types identified by Seurat.

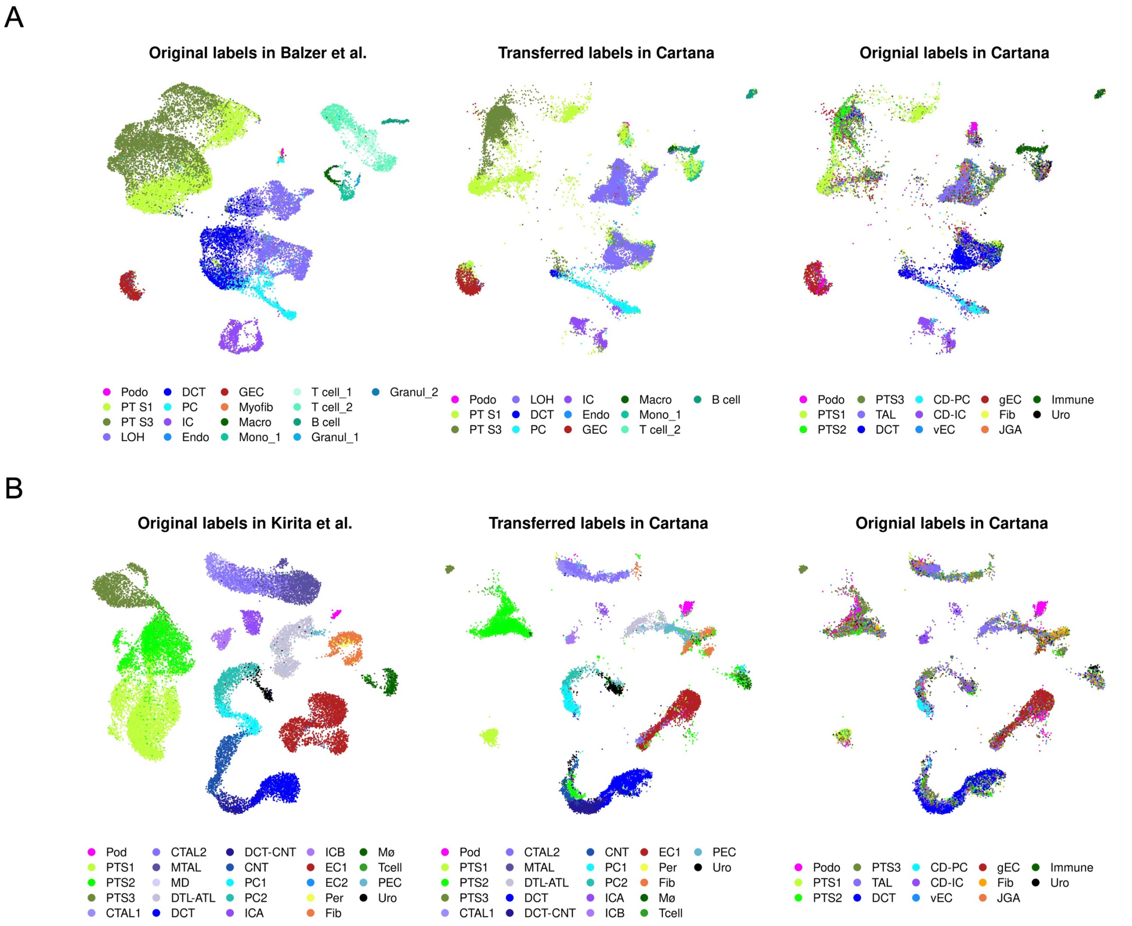

Supplementary Figure 8. Integrating Cartana data with public scRNA-seq and snRNA-seq Data. (**A**) Combined analysis of our spatial data and the scRNA-seq dataset from Balzer et al. (Nature Comms 2022). (**B**) Combined analysis of our spatial data and the snRNA-seq dataset from Kirita et al. (PNAS 2020). Cells are color-coded based on annotations from the original publications, our Cartana annotations, or predicted cell labels using Seurat label transfer derived from the original annotations.

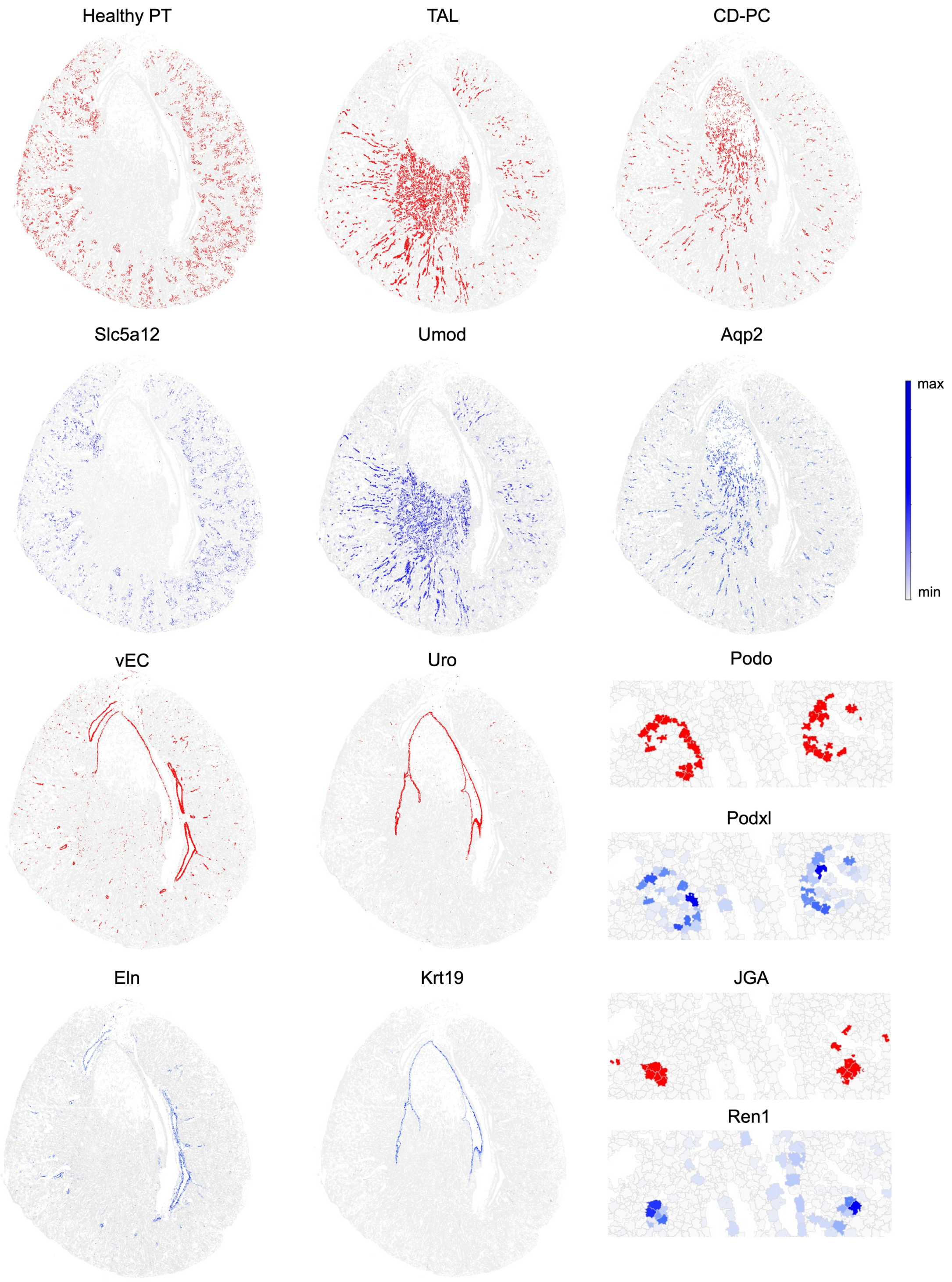

Supplementary Figure 9. Cell type annotations and anchor genes expression
Spatial distribution of the kidney cell types is consistent with the spatial expression of the anchor gene for each cell type.

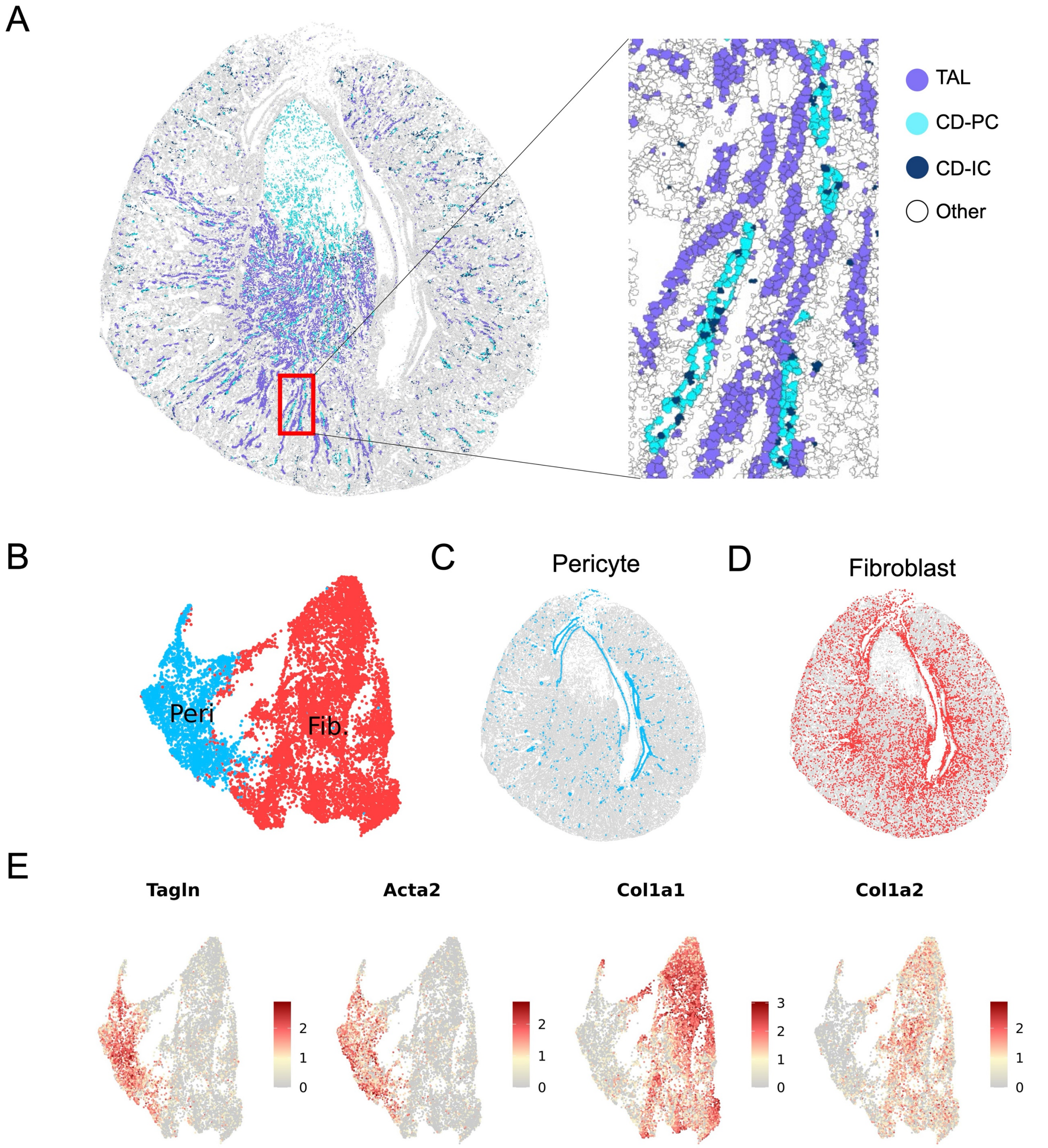

Supplementary figure 10. Spatial relationship of TAL, PC and IC cell types. (**A**) Spatial distribution of TAL, CD-PC and CD-IC in tissue. PC and IC cells are intermingled within the same tubule. (**B**) Subclustering the fibroblasts from the Day 2 IRI sample. UMAP showing the fibroblast subtypes. (**C**) Spatial distribution of the pericyte in the day 2 kidney. (**D**) Spatial distribution of the fibroblast. (**E**) Expression of the pericyte (Tagln and Acta2) and fibroblast genes (Col1a1 and Col1a2).

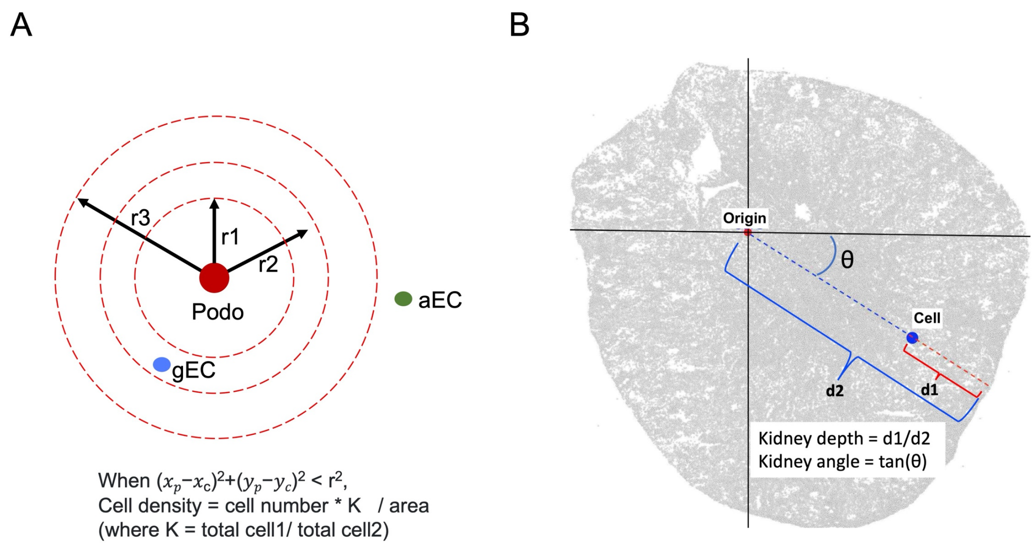

Supplementary Figure 11. Measurement of cell proximity and coordinate transformation. (**A**) A cell-centric approach was designed to measure cell-cell distance, whereby the density of a particular cell type within a given radius uses as a readout for the distance between that cell type and other cell types. A higher cell density indicates that the cells are closer to each other. (**B**) A novel coordinate system has been developed to re-define the spatial location of cells in the kidney. This system defines the position of each cell by two parameters: kidney depth, which measures the distance between the cell type and the kidney capsule, and kidney angle, which measures the angle of the cell's slope relative to the horizontal plane.

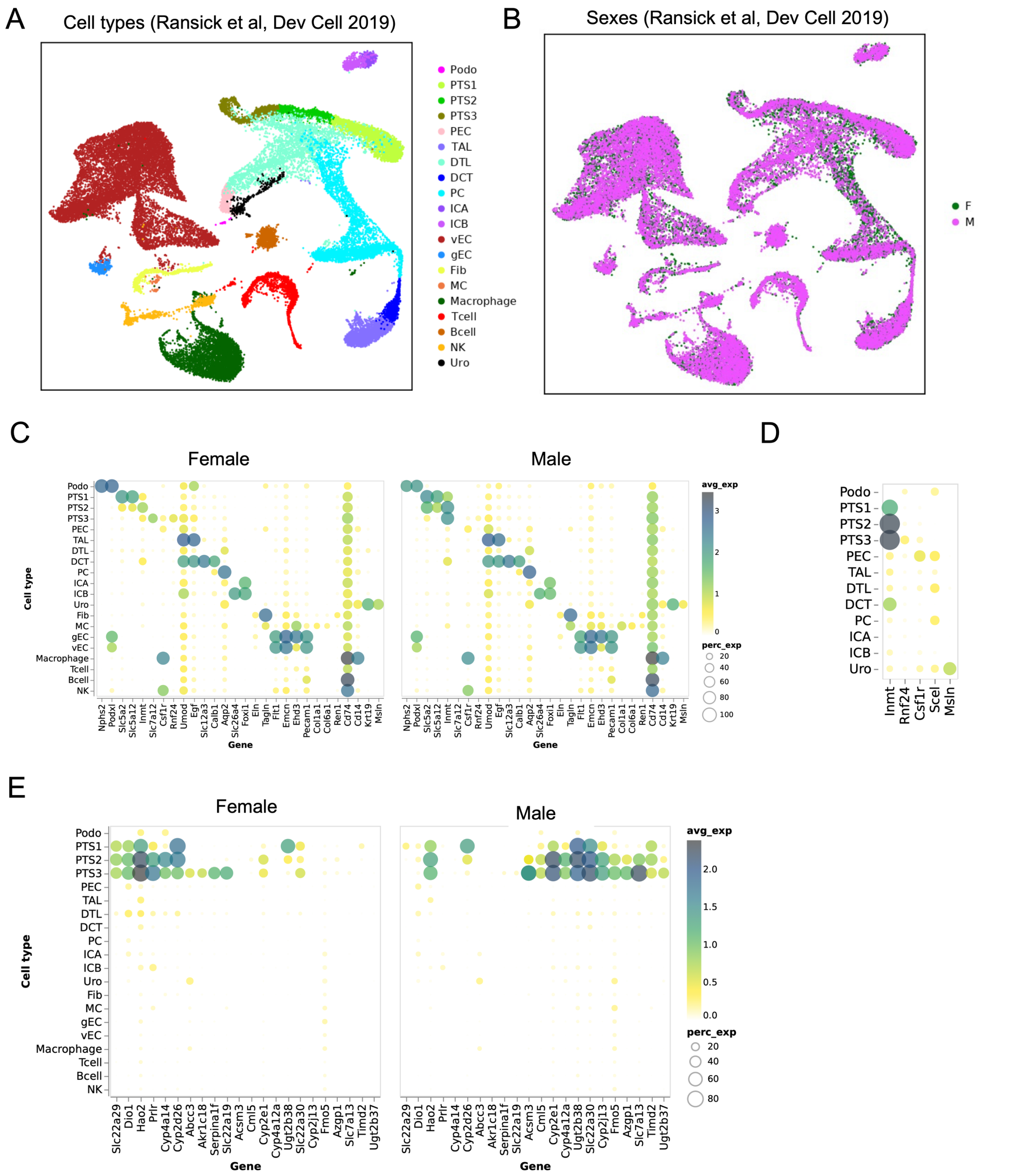

Supplementary Figure 12. Re-analysis of a scRNA-seq dataset on male and female kidneys. (**A**) Re-clustering of the public scRNA-seq dataset identified similar kidney cell types as reported. (**B**) The batch effect between male and female was successfully corrected. (**C**) Common known anchor genes to define the kidney cell types. (**D**) New marker genes identified from re-analysis. (**E**) Sex dimorphic genes for the proximal tubule in female or male kidney.

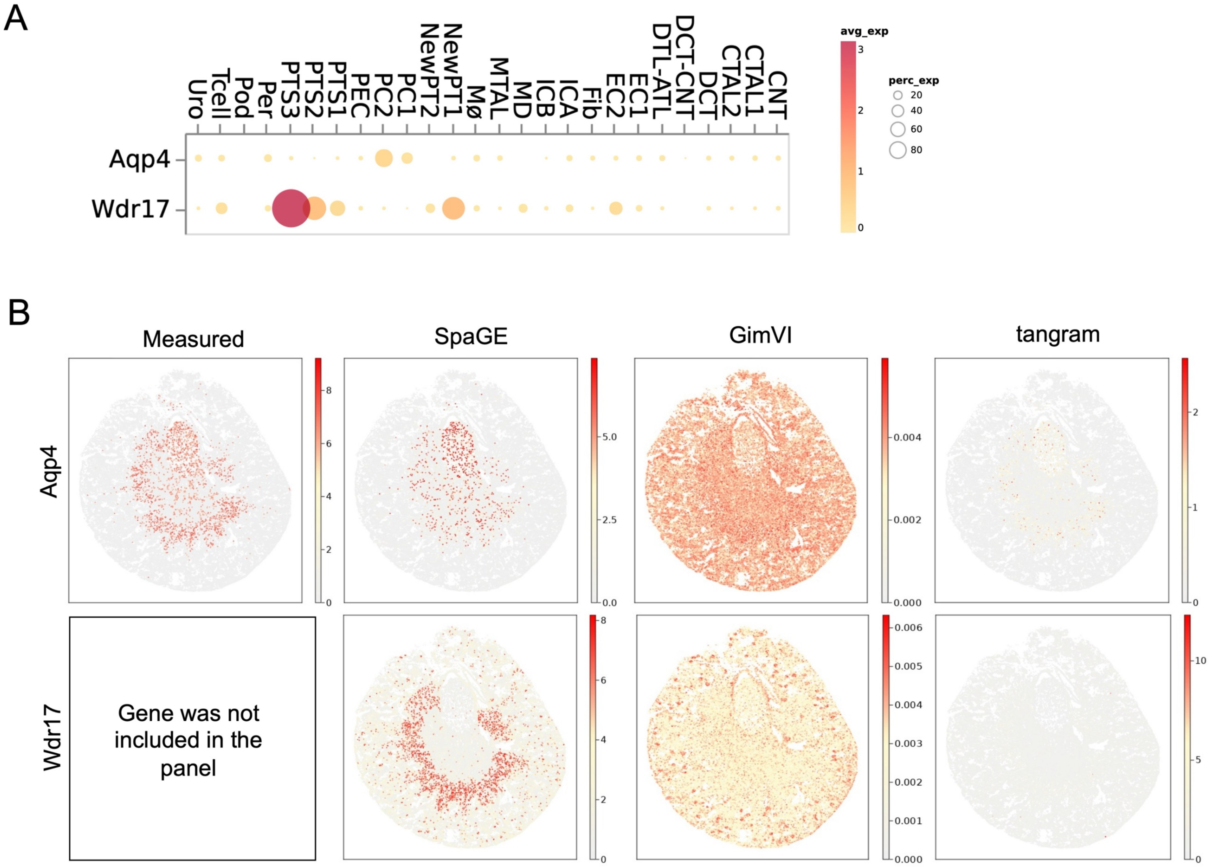

Supplementary Figure 13. Comparing various tools for spatial gene imputation. (**A**) Anchor genes for principal cells (Aqp4) and PTS3 cells (Wdr17) were selected from scRNA-seq data. Aqp4 was incorporated into our probe design, while Wdr17 was not included. (**B**) Visualization of the spatial expression of the Aqp4 and Wdr17 after gene imputation.

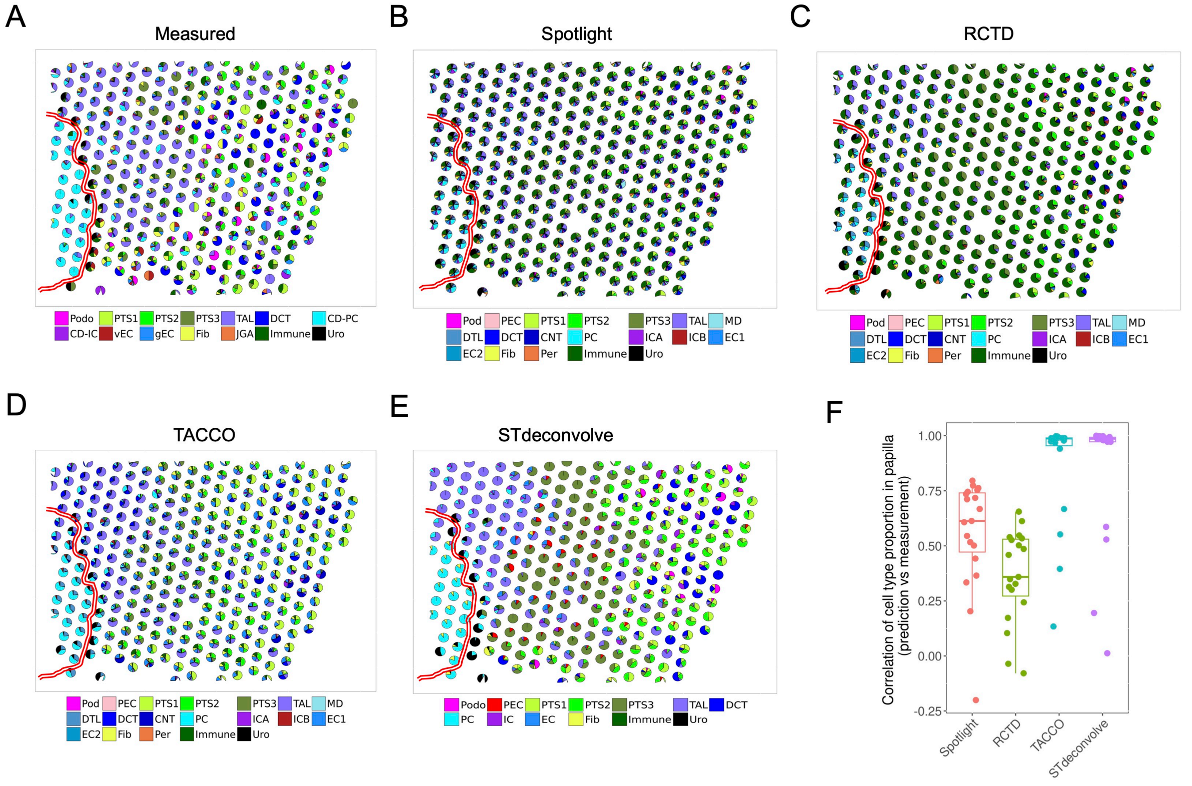

Supplementary Figure 14. Benchmarking various cell type deconvolution tools on Visium data. (**A**) Cell type proportion measured by dRNA HybISS. (**B** - **E**) Cell type proportion estimated by Spotlight (**B**), RCTD (**C**), TACCO (**D**) and STdeconvolve (**E**). (**F**) Pearson correlation between the cell type proportions obtained from direct measurement and computation-based deconvolution on the Visium spots from the papilla.

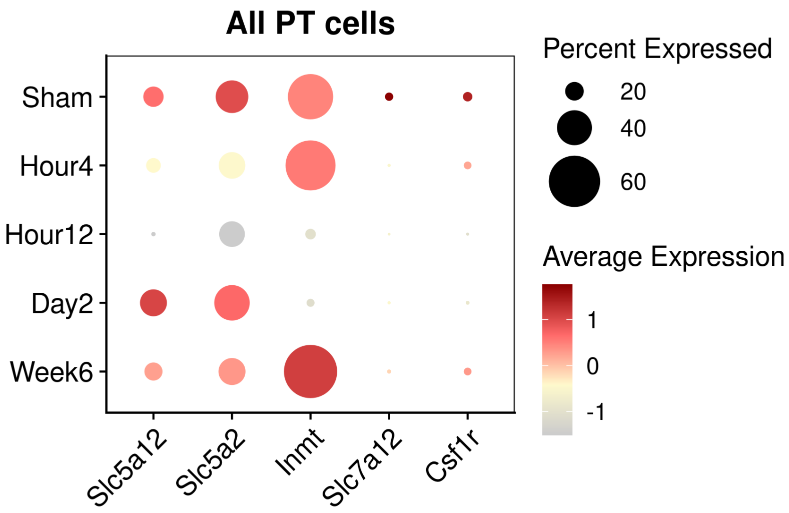

Supplementary Figure 15. Expression of PT subpopulation genes across timecourse. Dotplot showing the expression of PT S1 markers (Slc5a12 and Slc5a2), PT S2 Marker (Inmt) and PT S3 markers (Slc7a12 and Csf1r).

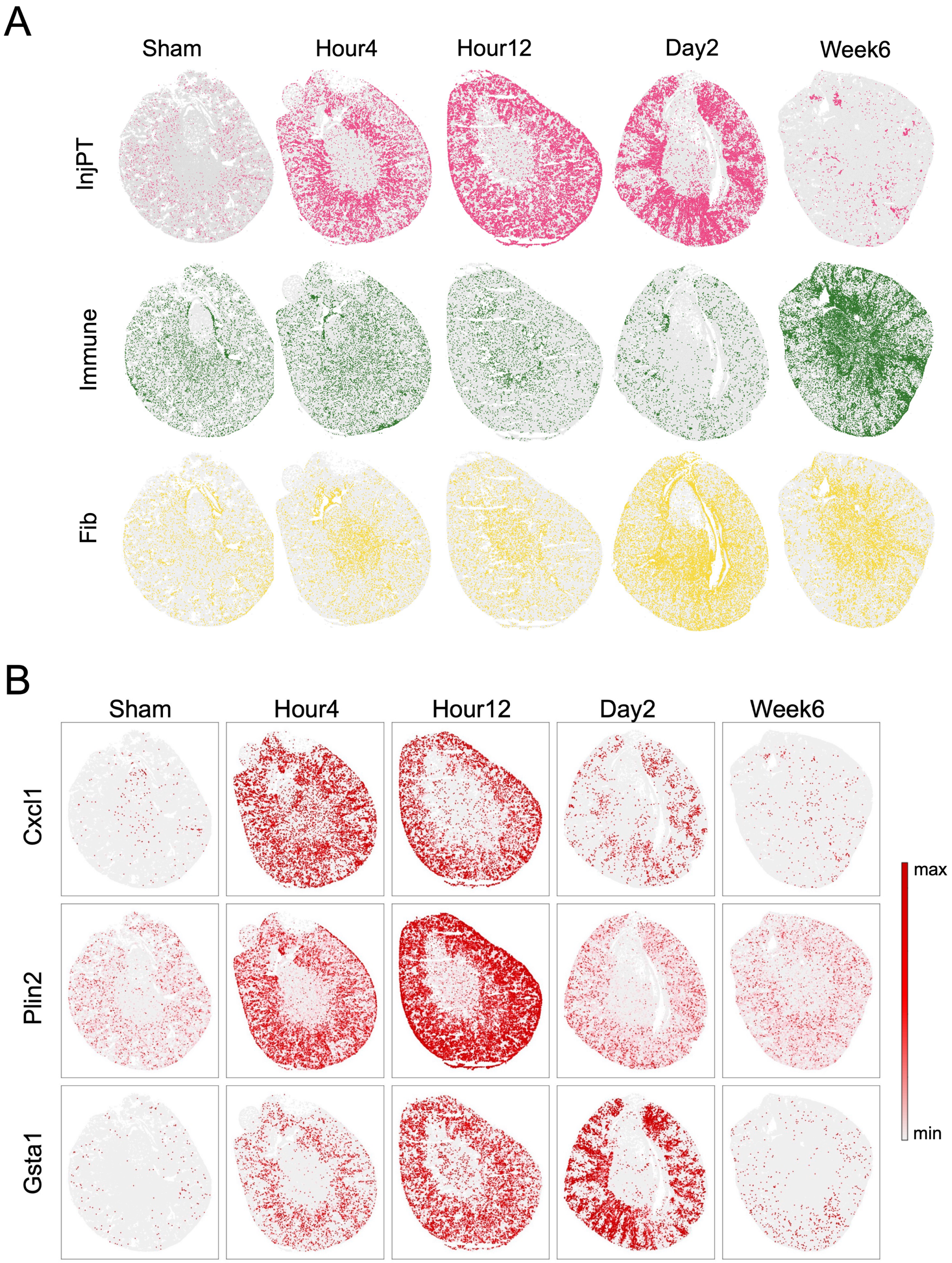

Supplementary Figure 16. Cell type distributions and gene expression across IRI timecourse. (**A**) Spatial distribution of the injPT, immune cells and fibroblast in all IRI time points. (**B**) Spatial expression of the IRI disease genes such as Cxcl1, Plin2 and Gsta1 across IRI time points.

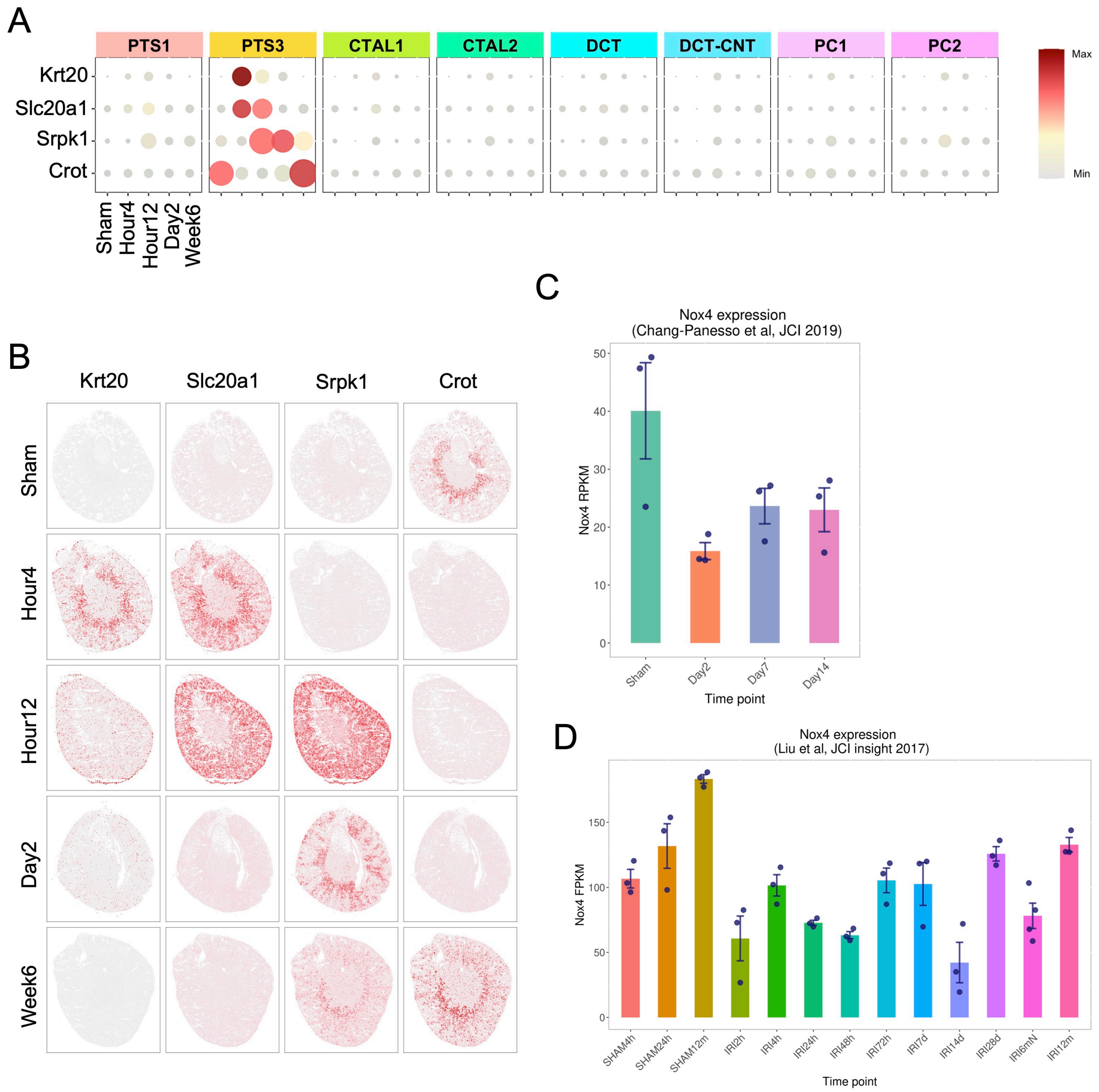

Supplementary Figure 17. Gene imputation to validate the new disease gene for AKI. (**A**) PTS3 specific disease genes identified from our previous snRNA-seq dataset. (**B**) Spatial distribution of these new disease genes using our time course dRNA HybISS dataset. Note that the expression values were imputed by SpaGE. (**C**) Expression of Nox4 from IRI bulk RNA-seq dataset from Chang-Panesso et al, JCI 2019. (**D**) Expression of Nox4 from IRI bulk RNA-seq dataset from Liu et al, JCI insight 2017.

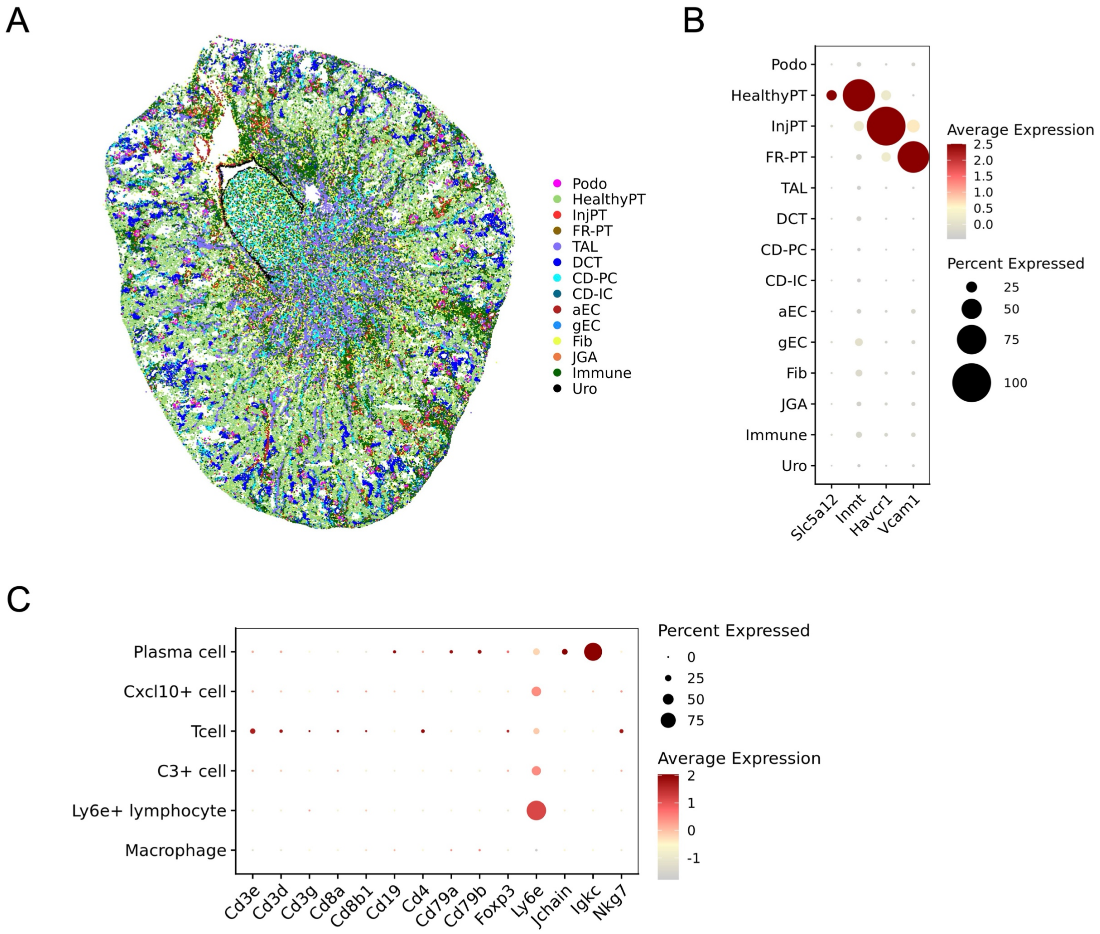

Supplementary Figure 18. Refinement of the cell type identification in the week 6 kidney. (**A**) Cells from the week 6 kidney were reclustered and colored by the cell type annotations. Three PT states were classified: healthy, injured and failed-repair PT. (**B**) Expression of the PT disease state specific markers. (**C**) Expression of the lymphocyte and neutrophil markers in the immune subpopulations at week 6.
