# Supplementary material for: High resolution spatial profiling of kidney injury and repair using RNA hybridization-based in situ sequencing
Source: Nat Commun. 2024 Feb 15;15:1396. doi: 10.1038/s41467-024-45752-8 (PMC10869771; doi:10.1038/s41467-024-45752-8)
Supplement: Supplementary file 3 — Reporting Summary [file 41467_2024_45752_MOESM3_ESM.pdf]

nature portfolio

Corresponding author(s): Benjamin D. Humphreys

Last updated by author(s): Jan 15, 2024

Reporting Summary

Nature Portfolio wishes to improve the reproducibility of the work that we publish. This form provides structure for consistency and transparency in reporting. For further information on Nature Portfolio policies, see our Editorial Policies and the Editorial Policy Checklist.

Statistics

For all statistical analyses, confirm that the following items are present in the figure legend, table legend, main text, or Methods section.

n/a	Confirmed	
☐	☒	The exact sample size (*n*) for each experimental group/condition, given as a discrete number and unit of measurement
☐	☒	A statement on whether measurements were taken from distinct samples or whether the same sample was measured repeatedly
☐	☒	The statistical test(s) used AND whether they are one- or two-sided<br>*Only common tests should be described solely by name; describe more complex techniques in the Methods section.*
☐	☒	A description of all covariates tested
☐	☒	A description of any assumptions or corrections, such as tests of normality and adjustment for multiple comparisons
☐	☒	A full description of the statistical parameters including central tendency (e.g. means) or other basic estimates (e.g. regression coefficient) AND variation (e.g. standard deviation) or associated estimates of uncertainty (e.g. confidence intervals)
☐	☒	For null hypothesis testing, the test statistic (e.g. *F*, *t*, *r*) with confidence intervals, effect sizes, degrees of freedom and *P* value noted<br>*Give P values as exact values whenever suitable.*
☒	☐	For Bayesian analysis, information on the choice of priors and Markov chain Monte Carlo settings
☒	☐	For hierarchical and complex designs, identification of the appropriate level for tests and full reporting of outcomes
☐	☒	Estimates of effect sizes (e.g. Cohen's *d*, Pearson's *r*), indicating how they were calculated

Our web collection on statistics for biologists contains articles on many of the points above.

Software and code

Policy information about availability of computer code

Data collection	dRNA HybISS data were generated by using a workflow provided by Cartana, part of 10x Genomics. The Visium data were collected using the Visium kit from 10x Genomics. The public scRNA-seq datasets were downloaded from Gene Expression Omnibus (GEO). Xenium and Visium datasets were downloaded from the 10x Genomics website.
Data analysis	A Julia package for downstream data analysis has been deposited in GitHub: https://github.com/TheHumphreysLab/CellScopes.jl. Scripts to reproduce the figures is deposited in our Github page: https://github.com/TheHumphreysLab/Spatial_analysis. We also used Seurat V5 (https://satijalab.org/seurat/articles/get_started_v5), Squidpy (https://squidpy.readthedocs.io/en/stable/), and Giotto (https://giottosuite.readthedocs.io/en/latest/) to produce the figures for benchmarking purpose. The raw fastq files for Visium data was processed by the SpaceRanger software provided by 10X Genomics. For cell segmentation, Baysor (https://github.com/kharchenkolab/Baysor) or Cellpose (https://cellpose.readthedocs.io/en/latest/) were used.

For manuscripts utilizing custom algorithms or software that are central to the research but not yet described in published literature, software must be made available to editors and reviewers. We strongly encourage code deposition in a community repository (e.g. GitHub). See the Nature Portfolio guidelines for submitting code & software for further information.

Data

Policy information about availability of data

All manuscripts must include a data availability statement. This statement should provide the following information, where applicable:

- Accession codes, unique identifiers, or web links for publicly available datasets
- A description of any restrictions on data availability
- For clinical datasets or third party data, please ensure that the statement adheres to our policy

All spatial transcriptomics data generated in this study have been deposited in the Gene Expression Omnibus (GEO) under accession number GSE227046 (https://www.ncbi.nlm.nih.gov/geo/query/acc.cgi?acc=GSE227046). The dRNA HybISS (Cartana) data are available under accession number GSE227044 (https://www.ncbi.nlm.nih.gov/geo/query/acc.cgi?acc=GSE227044). The Visium raw data can be accessed under accession number GSE227045 (https://www.ncbi.nlm.nih.gov/geo/query/acc.cgi?acc=GSE227045). Source data are provided with this paper. Public single cell RNA-seq data were collected from GEO with accession numbers: GSE180420, GSE139107, and GSE182256. The Xenium and Visium datasets for human kidney were downloaded from the 10x Genomics website (https://www.10xgenomics.com/resources/datasets?query=&page=1&configure%5BhitsPerPage%5D=50&configure%5BmaxValuesPerFacet%5D=1000)

Research involving human participants, their data, or biological material

Policy information about studies with human participants or human data. See also policy information about sex, gender (identity/presentation), and sexual orientation and race, ethnicity and racism.

Reporting on sex and gender	N/A
Reporting on race, ethnicity, or other socially relevant groupings	N/A
Population characteristics	N/A
Recruitment	N/A
Ethics oversight	N/A

Note that full information on the approval of the study protocol must also be provided in the manuscript.

Field-specific reporting

Please select the one below that is the best fit for your research. If you are not sure, read the appropriate sections before making your selection.

☒ Life sciences ☐ Behavioural & social sciences ☐ Ecological, evolutionary & environmental sciences

For a reference copy of the document with all sections, see nature.com/documents/nr-reporting-summary-flat.pdf

Life sciences study design

All studies must disclose on these points even when the disclosure is negative.

Sample size	No sample size calculation was performed. Samples from 6 mice were processed to obtain ~100,000 single cells per sample. The number of cells analyzed in this study was sufficient to draw the conclusions described in the manuscript.
Data exclusions	Cells with noisy transcript detection signal were removed by the Baysor cell segmentation algorithm. We further filtered the cells that have less than 5 transcripts detected.
Replication	Each individual cell analyzed in this spatial transcriptomics data represents a unique data point or a replicate. Statistical analysis is built on the thesecell-level replicates.
Randomization	Randomization is not relevant to this study.
Blinding	No blinding was used during data collection or analysis. It was not considered necessary for the study, because the measurements could not be affected by the operator.

Reporting for specific materials, systems and methods

We require information from authors about some types of materials, experimental systems and methods used in many studies. Here, indicate whether each material, system or method listed is relevant to your study. If you are not sure if a list item applies to your research, read the appropriate section before selecting a response.

Materials & experimental systems

n/a	Involved in the study
☐	☒ Antibodies
☒	☐ Eukaryotic cell lines
☒	☐ Palaeontology and archaeology
☐	☒ Animals and other organisms
☒	☐ Clinical data
☒	☐ Dual use research of concern
☒	☐ Plants

Methods

n/a	Involved in the study
☒	☐ ChIP-seq
☒	☐ Flow cytometry
☒	☐ MRI-based neuroimaging

Antibodies

Antibodies used	Nox4 antibody was purchased from ThermoFisher (#PA5-95083). The Havcr1 antibody was from R&D Systems (#AF1817). Secondary antibodies Alexa Fluor® 488 anti-rabbit (711-545-152) was from Jackson ImmunoResearch and Alexa Fluor® 568 anti-goat (A11057) was purchased from Fisher Scientific.
Validation	The manfacturer website for Nox4 validation is here: https://www.thermofisher.com/antibody/product/NOX4-Antibody-Polyclonal/PA5-95083?imageId=561496.<br>Havcr1 is here: https://www.rndsystems.com/products/mouse-tim-1-kim-1-havcr-antibody_af1817?gclid=CjwKCAjw7oeqBhBwEiwALyHLM-f1yZxcxuLMMVW1s761gDiwlk2vhp_dum2jQ8l_JGOFgjfhw3AvwhoCLXIQAvD_BwE&gclsrc=aw.ds.

Animals and other research organisms

Policy information about studies involving animals; ARRIVE guidelines recommended for reporting animal research, and Sex and Gender in Research

Laboratory animals	All in vivo experiments were performed on 8- to 10-week-old C57BL6/J male mice from The Jackson Laboratory. Experiments and housing guidelines were executed in accordance with the Animal Care and Use Committee at Washington University in St. Louis. Mice were maintained on ad libitum food and water in a 12-hour light:dark cycle. The mouse housing room was maintained at humidity 30-70% and temperature 20-26°C (68-79°F).
Wild animals	Wild animals were not included.
Reporting on sex	Both male and female were included in this study.
Field-collected samples	Not applicable.
Ethics oversight	Experiments and housing guidelines were executed in accordance with the Animal Care and Use Committee at Washington University in St. Louis. Mice were maintained on ad libitum food and water in a 12-hour light:dark cycle.

Note that full information on the approval of the study protocol must also be provided in the manuscript.

Plants

Seed stocks	*Report on the source of all seed stocks or other plant material used. If applicable, state the seed stock centre and catalogue number. If plant specimens were collected from the field, describe the collection location, date and sampling procedures.*
Novel plant genotypes	*Describe the methods by which all novel plant genotypes were produced. This includes those generated by transgenic approaches, gene editing, chemical/radiation-based mutagenesis and hybridization. For transgenic lines, describe the transformation method, the number of independent lines analyzed and the generation upon which experiments were performed. For gene-edited lines, describe the editor used, the endogenous sequence targeted for editing, the targeting guide RNA sequence (if applicable) and how the editor was applied.*
Authentication	*Describe any authentication procedures for each seed stock used or novel genotype generated. Describe any experiments used to assess the effect of a mutation and, where applicable, how potential secondary effects (e.g. second site T-DNA insertions, mosiacism, off-target gene editing) were examined.*
